# Genomic Characterization and Molecular Epidemiology of Tusaviruses and Related Novel Protoparvoviruses (Family *Parvoviridae*) from Ruminant Species (Bovine, Ovine and Caprine) in Hungary

**DOI:** 10.3390/v17070888

**Published:** 2025-06-24

**Authors:** Fruzsina Tóth, Péter Pankovics, Péter Urbán, Róbert Herczeg, Ervin Albert, Gábor Reuter, Ákos Boros

**Affiliations:** 1 Department of Medical Microbiology and Immunology, Medical School, University of Pécs, H-7624 Pécs, Hungary; toth.fruzsina1@edu.pte.hu (F.T.); pankovics.peter@pte.hu (P.P.); reuter.gabor@pte.hu (G.R.); 2 János Szentágothai Research Centre, Bioinformatics Research Group, Genomics and Bioinformatics Core Facility, University of Pécs, H-7624 Pécs, Hungary; urban.peter@pte.hu (P.U.); herczeg.robert@pte.hu (R.H.); 3 Department of Pathology, University of Veterinary Medicine Budapest, H-2225 Üllő, Hungary; albert.ervin@univet.hu; 4 Institute of Metagenomics, University of Debrecen, H-4030 Debrecen, Hungary

**Keywords:** parvovirus, protoparvovirus, recombination, ruminant, epidemiology, ovine, caprine, cattle, NGS, phylogenetics

## Abstract

Tusavirus 1 of species *Protoparvovirus incertum 1* (family *Parvoviridae*) was first identified in humans and later in small ruminants (caprine and ovine). This study reports the full-length coding sequences (~4400–4600 nt) of three novel tusavirus-related protoparvoviruses from ovine (“misavirus”, PV540792), for the first time bovine (“sisavirus”, PV540793) and subsequently from caprine (“gisavirus” PV540850/51) fecal samples, using next-generation sequencing (NGS) and PCR techniques. Their NS1, VP1 and VP2 proteins shared 61–63% amino acid identities with each other and with tusaviruses, suggesting these three viruses belong to three novel species in the genus *Protoparvovirus*. Phylogenetic analyses placed them with tusaviruses on a separate main branch, implying a shared origin among these most likely ruminant protoparvoviruses. A small-scale epidemiological investigation on 318 ruminant enteric samples using novel generic NS1 primers found misavirus in 14/51 (27.5%) ovine and sisavirus in 19/203 (9.4%) bovine samples from multiple Hungarian farms. Tusavirus was present in 5/51 (9.8%) ovine and 15/62 (24.2%) caprine samples, all from one farm. The highest prevalences for all three viruses were found in animals aged 2–12 months, though sporadic cases were also found in other age groups. Partial NS and VP sequence-based phylogenetic trees showed virus-specific lineages for misa-, sisa-, gisa- and tusaviruses, with various strains forming sub-lineages. These findings suggest the presence of multiple genotypes and/or members of additional species, which was supported by a VP sequence-based hierarchical cluster analysis. The study’s viruses were mostly phylogenetically separated by host; however, two bovine sisavirus strains with diverse phylogenetic localizations in the NS (belonging to bovine sisaviruses) and VP1 trees (distantly related to ovine misaviruses) could indicate previous (interspecies?) recombination events.

## 1. Introduction

Parvoviruses are a genetically highly diverse group of small, non-enveloped viruses with 4–6 kb. long, linear single-stranded DNA (ssDNA) genomes, flanked by 3′ and 5′ untranslated regions with partially double-stranded DNA sections forming hairpin structures, creating inverted terminal repeats (ITRs) [1,2]. The family *Parvoviridae* consists of three subfamilies, *Densovirinae* (infecting invertebrates)*, Hamaparvovirinae* (infecting both invertebrates and vertebrates) and *Parvovirinae* (infecting vertebrates), containing 11, 5 and 11 genera, respectively [1,3]. The non-segmented genomes of viruses in the genus *Protoparvovirus,* subfamily *Parvovirinae,* have three major open reading frames (ORFs) encoding three proteins, a non-structural (NS1), replication initiator protein and capsid or viral proteins (VP1 and VP2), although in certain protoparvoviruses like cuta- and bufaviruses additional minor ORFs were also presumed [1,2,4].

The polarity of the packaged mature viral ssDNA varies in the subfamily *Parvovirinae,* based on the viral hairpin sequences flanking the ends of the genome, which affect the cleavage of the genome parts during replication. Usually, both polarities are present in the virions but in different proportions varying by genus [5,6,7].

Parvoviruses, based on their short, single-stranded DNA genomes, have a high intrinsic mutation rate, which, paired with positive selective pressure, could result in a possible genomic substitution rate of around 10^−4^ per site per year, making them more like RNA viruses, rather than other DNA viruses, in this respect [8,9]. Besides their high mutation rate, some parvoviruses (like canine parvovirus, CPV) can frequently generate recombinants between variants or closely related viruses [10,11,12,13], while others tend to generate multiple genotypes (like human and canine bufaviruses), similar to what is found in RNA viruses [14,15,16,17].

Protoparvoviruses in animals can cause a range of conditions, from asymptomatic infection (Minute Virus of Mice) to immunosuppression, hemorrhagic diarrhea (CPV) or abortion and infertility (*Protoparvovirus ungulate 1*, *Porcine parvovirus 1*), sometimes with lethal outcomes, resulting in serious economic losses and welfare problems in different domestic animal populations [1,18,19,20,21]. Meanwhile little is known about other recently identified human protoparvoviruses like tusaviruses (TusaVs) of species *Protoparvovirus incertum 1,* which were first discovered in 2014 in a fecal sample of a Tunisian child with diarrheic symptoms [22]. Since its discovery TusaV DNA has been detected in human fecal samples only a handful of times, with a low prevalence of 1.82% (2/1098) in adults from Finland and 0.52% (2/3837) in chronic patients from China [23,24]. The measured seroprevalence is low, ranging from 0% in adults and children with different health conditions from Finland, Kenya, Italy, Iran, Iraq and the USA to 0.44% in Finnish children and 0.80% in Finnish transplant patients [23,25,26,27,28]. However, TusaVs have been detected in small ruminants (caprine and ovine) in Hungary [29] with a relatively high enteric prevalence (17.8% and 25.5%, respectively) as well as in the UK [30], suggesting that TusaVs may primarily infect ruminants and have zoonotic potential, but unlike other protoparvoviruses, the clinical picture of TusaV infections remains incomplete.

Here we report the presence of multiple newly detected, tusavirus-related protoparvoviruses from ruminant species (cattle, ovine and subsequently in caprine) with a relatively high prevalence, including potential recombinants, as well as additional caprine tusaviruses, related to ovine and human strains in Hungary using viral metagenomics, next-generation sequencing and various PCR and Sanger sequencing techniques.

## 2. Materials and Methods

### 2.1. Background Information on Samples, Animals and Farms

**Figure 1:**
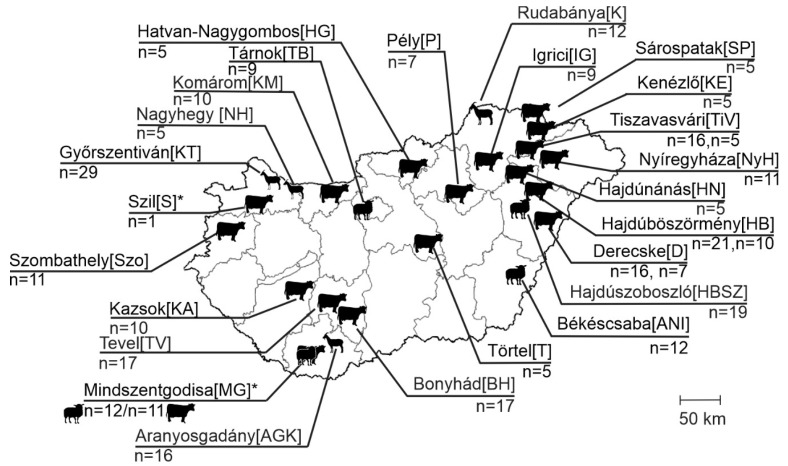
Geographical locations of investigated farms and farm identifications [IDs] in Hungary, as well as the number of collected enteric samples (*n* = x). Animal species are marked with silhouettes. *: Localization of index farms from where the samples used for next-generation sequencing originated.

In this study, a total of 318 enteric samples from different domestic ruminant species, ovine (*n* = 52), caprine (*n* = 62) and bovine (*n* = 204), were used, which were collected from 125 diarrheic and 193 asymptomatic animals from 25 geographically distant farms across Hungary between 2008 and 2024 (Table S1, Figure 1). Most of the ovine and caprine farms and one bovine farm (Derecske), were extensively managed (except for Győrszentiván), where animals of all age groups were held together, freely, outside during the day and inside during the night. In contrast, most of the bovine farms, along with the one caprine farm, were intensively managed, with animals strictly indoors, separated by age groups and, in the case of the bovine held in separate stalls. Most of the farms were limited to one species, but there was one mixed farm, where ovine and bovine were held together in Mindszentgodisa (Figure 1). For epidemiological investigations, the sampled animals were retrospectively classified into three arbitrary age groups—group I: <2-month-old, group II: 2–12-month-old, group III: >12-month-old (Table S1).

### 2.2. Viral Metagenomics and Next Generation Sequencing

A detailed description of the viral metagenomics (VM), next-generation sequencing (NGS) and bioinformatics pipeline applied in this study can be found in our previous report [31]. Briefly, fecal samples were suspended in 0.1M sterile PBS to obtain ≈40 *v*/*v*% suspensions. The fecal suspensions were then centrifuged in 8000× *g* for 5 min, and supernatants were filtered through Ø: 0.45 µm PES membrane filters (Millipore/Merck, Burlington, MA, USA). The unprotected nucleic acids were digested with a DNase/RNase cocktail. Nuclease-treated fecal filtrates of three, less than 1-year-old diarrheic ovine were mixed with a volume ratio of 1:1 to create a sample pool (MGJ-pool). Besides the pooled ovine sample, a single enteric sample from 4–5-month-old diarrheic cattle, and subsequently a ≈12-month-old goat, was also investigated by VM-NGS in a separate reaction/run. The total nucleic acid (NA) isolations from the pooled and single samples were performed using the Quick RNA Kit (Zymo Research, Irvine, CA, USA) according to the general protocol of the kit. After the library’s construction, the samples were run on a NovaSeq X Plus (Illumina, San Diego, CA, USA) platform. For the identification of the viruses, the generated raw sequencing reads were analyzed by an “in-house” assembled bioinformatics pipeline, which includes adapter filtration, a quality check and Kaiju and Diamond aligner software with the use of the NCBI RefSeq databases. For the de novo assembly of the filtered and quality-checked reads and for the mapping of selected reads to a reference genome, we used Geneious Prime software ver.2024.1.1 (Biomatters, Auckland, New Zealand). The reference genome was selected based on the results of BLASTx/n searches of selected reads/contigs.

### 2.3. Genome Determination and Screening Reactions Using PCR and Sanger Sequencing Methods

The total nucleic acid was isolated from individual fecal samples re-suspended in 0.1M sterile PBS using a NucleoSpin RNA Kit (Macherey-Nagel, Düren, Germany) following the manufacturer’s instructions but without the DNase treatment step.

The complete coding sequences of the study viruses were determined using read-specific and generic oligonucleotide primer pairs by various PCR techniques (conventional, nested and 5′ RACE PCR). Generic primers were designed based on the alignment of the most closely related (by BLAST searches, ver. BLAST+ 2.16.0) tusavirus sequences downloaded from the GenBank database. The reagents and conditions used for conventional and nested PCR reactions were the same as described in our previous study [32]. The 5′ regions of the sense and antisense genomes were determined by a modified version of the 5′RACE-PCR method described in the study of Jones and co-workers [33]. Briefly, the 5′ ends of the viral genomes of ovine and bovine protoparvoviruses were amplified linearly using virus-specific reverse (for sense genomes in the sample) and forward primers (for antisense genomes in the sample). The linearly amplified products were then purified by ethanol precipitation and ligated with an ssDNA Adapter using T4 RNA ligase (Thermo Fisher, Waltham, MA, USA) and a protocol described previously [34,35]. The 5′ ends were amplified in a semi-nested PCR setup with virus-specific and Adapter-specific primers. Acquired PCR products were sequenced directly using primer walking with a BigDye Terminator v.1.1 Cycle Sequencing Kit (Thermo Fisher, Waltham, MA, USA) and run on an automated ABI 3500 Genetic Analyzer (Applied Biosystems, Hitachi, Tokyo, Japan). The sequencing data were analyzed using Chromas ver.2.6.76 (Technelysium, South Brisbane, Australia) and Geneious Prime ver2024.1.1 (Biomatters, Auckland, New Zealand). The primer sequences in this study used for genome determination reactions can be made available upon request.

For epidemiological investigations and virus typing, separate generic primer sets (Table 1) targeting the NS and VP regions in two different, subsequent nested PCR reactions were designed based on nucleotide alignments of the study ovine and bovine protoparvoviruses and all available tusavirus sequences downloaded on 22 May 2024, from the GenBank database.

### 2.4. In Silico Sequence, Phylogenetic and Recombination Analyses

Multiple sequence alignments used for the phylogenetic analyses, sequence comparisons and primer designs were generated by the Multiple Sequence Comparison by Log-Expectation (MUSCLE) web tool [36]. For genomes’ assembly, analyses and pairwise nucleotide (nt) and amino acid (aa) identity calculations, we used GeneDoc ver.2.7 [37] and Geneious Prime ver2024.1.1 software. Potential open reading frames were predicted by the ORFfinder of NCBI (https://www.ncbi.nlm.nih.gov/orffinder/, accessed on 19 November 2024) and from alignments with closest known relatives.

The phylogenetic trees based on nt and deducted aa sequence alignments were constructed using the W-IQ-TREE interface and iTOL for visualization and annotation [38,39] and MEGA ver11 software using various models indicated in the figure legends [40].

Distance plots were calculated with RDP5 software using the “Similarities” model with a window size of 100 nt and a step size of 5 nt [12]. SimPlot ver. 3.5.1 and RDP5 software were used for bootscanning analyses (recombination detection) [41,42].

We also predicted putative splicing sites for the expression of the VP1 protein with the use of the Spliceator online tool (https://www.lbgi.fr/spliceator/, accessed on 19 November 2024) and the alignments of other protoparvovirus sequences with previously determined potential splicing sites [43,44].

The hierarchical cluster analysis was performed using a “cluster_analysis” script written in RStudio, version (RStudio 2024.09.0 + 375) using the “ape”, “cluster”, “cowplot”, “factoextra”, “ggrepel”, “gridExtra”, “msa”, “plotly”, “randomcoloR” and “seqinr” R packages [45]. The plots generated from the analysis in RStudio were post-edited using CorelDRAW Standard ver. 2022 and InkScape ver. 1.3.2 software. The partial VP2 nucleotide sequences were aligned using ClustalW, and distances were calculated. Hierarchical clustering with a complete linkage was performed. Optimal groups were determined using silhouette, Elbow/WSS, and GapStatistic methods from the factoextra package’s fviz_nbclust function, considering genetic distances. Dim1 and Dim2 percentages indicate the proportion of total variance explained by each axis, with higher percentages capturing more variability [46,47].

## 3. Results

### 3.1. Genome Analysis of Ovine and Bovine Protoparvoviruses

First a pool of three fecal samples of diarrheic ovine from Mindszentgodisa (individual sample IDs: MG-J1; -J4; -J6) and a single enteric sample of diarrheic cattle from Szil (sample ID: S2794) were subjected to VM-NGS (NovaSeq X Plus, Illumina). By the bioinformatic analyses of the acquired NGS data a total of 9 reads from the ovine pool and 51 reads from the bovine sample (from which a total of 6 contigs could be de novo assembled) were identified as protoparvovirus-related sequences. The acquired reads were aligned to the Tusavirus 1 strain Tu491 (KJ495710/NC_075988) as the closest relative identified by BLASTn/x searches. With the use of read-specific and generic oligonucleotide primers, various PCR methods and Sanger sequencing techniques, from the ovine sample (sample ID: MG-J1) a 4693 nt long, and from the bovine sample (S2794) a 4806 nt long, complete coding protoparvovirus genome was determined. These ovine and bovine protoparvoviruses were tentatively named as “misavirus” (MisaV strain ovine/MisaV/MG-J1/HUN/2022, PV540792) and “sisavirus” (SisaV strain bovine/SisaV/S2794/HUN/2021, PV540793) based on the origins of the samples: Mindszentgodisa and Szil and stool-associated virus, respectively.

The complete coding sequences (CDSs) of MisaV and SisaV showed 63.15% and 61.94% nt identities to the Tusavirus 1 strain Tu491, respectively, as the closest match identified by BLASTn search. MisaV and SisaV were found to have the open reading frames (ORFs) characteristic of members of the subfamily *Parvovirinae*, encoding a non-structural protein (NS1) and viral or capsid proteins (VP1/VP2), which were flanked by 5′- and 3′-untranslated regions.

**Figure 2:**
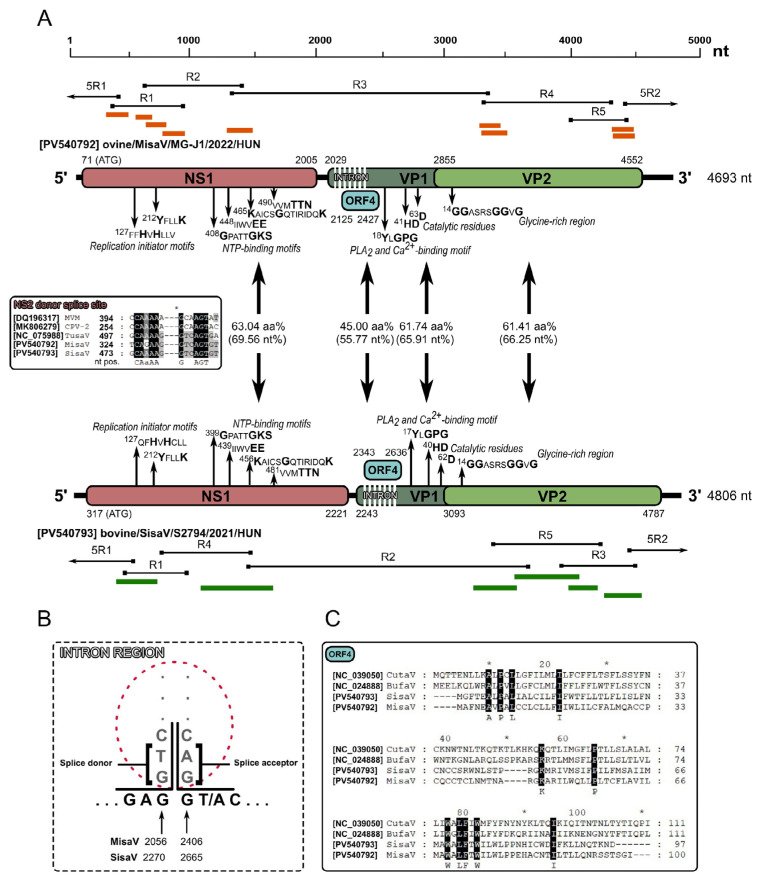
(**A**) Schematic representation of the genome organization of the study’s protoparvoviruses, misavirus and sisavirus. The genome regions characteristic of parvoviruses, the non-structural protein (NS1), the viral/structural proteins (VP1, VP2) and the hypothetical ORF4 are illustrated with pink, dark green, light green and light blue, respectively. The start and end nucleotide (nt) positions of the protein-coding genome regions are illustrated above (misavirus) or below (sisavirus) the specific regions, along with the illustrations of the NGS reads and contigs (orange and green bars) and the overlapping PCR products from the genome acquisition (black bars with black squares at the end, indicating the oligonucleotide primers) and 5’RACE reactions (5R1-2, black bars, with black arrows). The lengths of the acquired genomes are marked on the right side of the genomes. The pairwise amino acid (upper numbers) and nucleotide (lower numbers, in brackets) sequence identity values (%) of different genomic regions are found between the corresponding boxes. The positions of conserved parvoviral amino acid (aa) motifs of the study strains are indicated with the first amino acid position of the motif and the letters in **bold,** illustrating the most conserved amino acids between parvoviruses. The nt alignment of the presumed NS2 donor splice site is found in the insert of panel A. CPV-2: canine parvovirus 2, MVM: minute virus of mice. (**B**) Theoretical splicing for the expression of the VP1 of the study strains, indicating the donor and acceptor site positions and the cleaved intron region. (**C**) Alignment of the amino acid sequences of the hypothetical ORF4 protein of the study strains with the most closely related strains of cuta-, (CutaV) and bufaviruses (BufaV) identified by BLASTp searches.

The 644 aa and 634 aa long NS1 protein-encoding ORF1 started with a potential start codon in positions 71/317 in MisaV/SisaV, which was found in an optimal Kozak context [48,49]. We identified two replication initiator (endonuclease) motifs (xx**H**u**H**xxxx and **Y**xxx**K,** where x represents any amino acids, u represents hydrophobic amino acids, and letters in **bold** show conserved amino acids) in the NS1 proteins [50] (Figure 2A). The NS1 proteins of MisaV/SisaV also contained a viral helicase with NTP-binding motifs, Walker loops A (**G**xxxx**GKT**/**S**), B (xxxx**EE**), B’ (**K**xxxx**G**xxxxxxx**K**) and C (uux**TTN**) [51,52] (Figure 2A). These were almost identical in the two viruses, but the aa positions vary based on a deletion in the NS1 encoding region of the SisaVs. The NS1 proteins showed 63.04% and 62.14% aa identities to the corresponding protein of the reference TusaV-1 sequence (KJ495710/NC_075988), respectively (Table 2).

The VP1 capsid-encoding ORF could start from a translation initiation codon found in a weak Kozak context at nt positions 2029 in MisaV and 2243 in SisaV. Based on the results of Spliceator, as well as the manual sequence analyses, a theoretical splicing donor/acceptor site conserved among protoparvoviruses [1,4], can be identified at nt positions 2056/2406 and 2270/2665 of misa-/sisavirus resulting in the expression of 723/715 aa long VP1 capsid proteins (Figure 2A,B). An additional most likely donor site (was also identified at nt positions 330/479 in the NS1 encoding ORF of MisaV/SisaV (insert of Figure 2A) which could be the splice site of NS2, but the potential acceptor sites cannot be predicted with great certainty. Besides VP1, a shorter capsid-encoding ORF (VP2) with a potential start codon at nt positions 2855 and 3093 of misa-/sisavirus can also be identified, encoding a 565/564 aa long VP2 capsid protein, respectively (Figure 2A).

Like in other protoparvoviruses (and the members of subfamily *Parvovirinae*), the phospholipase A_2_ (PLA_2_) was also identifiable at the N-termini of the VP1 of the study viruses with the conserved Ca^2+^-binding site **Y**x**GPG** and catalytic residues **HD** and **D** [14,15,16,22,53,54] (Figure 2A). Near the N-terminal part of the VP2 protein, a glycine-rich region, ^14^**GG**ASRS**GG**V**G**, also presents similarly to what is found in other protoparvoviral VP2 sequences [4,14,22,54]. The nt/aa sequence identity values of the main viral proteins (NS1, VP1 and VP2) to the reference TusaV-1 sequence are found in Table 2.

Sequence analyses of misa- and sisaviruses revealed the presence of a potential middle ORF (ORF4), which encodes a 100 and 97 aa long hypothetical protein of unknown function with no significant hit found in the GenBank database by BLASTp searches. These hypothetical proteins share a low sequence identity of 45% with each other, as well as with other related protoparvoviruses, which exhibit known ORF4 protein identities ranging from 20.72 to 25.22%, such as bufa- and cutaviruses (Figure 2C).

**Figure 3:**
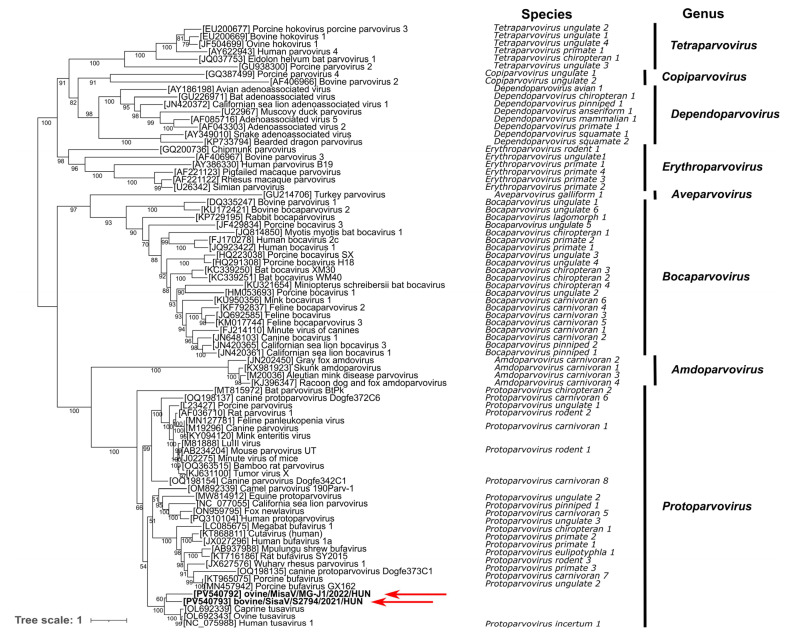
Phylogenetic relationship of the study viruses (marked with **bold** and red arrows), most closely related tusaviruses and representative members of the subfamily Parvovirinae, based on the amino acid sequences of the NS1 protein. The tree was generated by the maximum likelihood method with an LG + F + I + G4 model with 1000 bootstrap (BS) replicates with IQTree, visualized with iTOL. Only BS values of 50 or more are indicated at the nodes.

**Figure 4:**
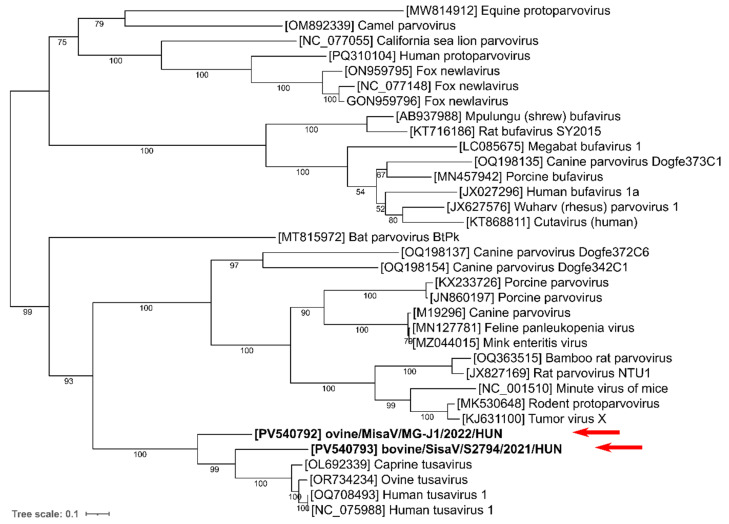
Phylogenetic analysis of the study viruses (marked with **bold** and red arrows), the most closely related tusaviruses and representative members of genus Protoparvovirus, based on the amino acid sequences of the full-length VP2 viral protein. The tree was generated by the maximum likelihood method with an LG+F+I+G4 model with 1000 bootstrap (BS) replicates with IQTree, visualized with iTOL. Only BS values of 50 or more are indicated at the nodes.

Phylogenetic analyses of the NS1 and VP1 proteins indicate that both study strains branch together with the tusaviruses in the same main lineage among the known protoparvoviruses but form two separate sub-lineages (Figure 3 and Figure 4).

### 3.2. Epidemiological Investigations of Ruminant Protoparvoviruses

To investigate the prevalences and genetic diversities of misa- and sisaviruses, as well as the related tusaviruses, a small-scale epidemiological investigation was conducted on a total of 316 archived enteric samples from ovine (*n* = 51), caprine (*n* = 62) and cattle (*n* = 203), using generic primer pairs targeting the NS (for screening reactions) and VP regions (for typing reactions) of the MisaV/SisaV, as well as all the known tusaviruses, and used in nested PCR. Besides the index samples (MG-J1 and S2794), a total of 19/51 (37.3%) ovine, 15/62 (24.2%) caprine and 19/203 (9.4%) bovine samples were positive in NS screening PCR reactions. Note that 50.0%, 60.0% and 85.0% of the positive ovine, caprine and bovine samples showed positivity only in the second PCR round (Table S1). Due to the broad detection range of the applied generic NS primer pairs (Table 1), as well as the identical PCR product sizes of all three viruses, all products had to be sequenced for proper identification. A total of 53 s-round PCR products were sequenced. The acquired 328 nt long NS sequences showed a >72.7% pairwise nt identity to each other. The PCR-positive samples originated from 3/4, 1/4 and 7/18 of investigated ovine, caprine and bovine farms, respectively (Table 3 and Table S1).

Based on sequence comparisons and individual BLASTn analyses, 14/19 and 5/19 of the acquired NS sequences from ovine belong to MisaV and TusaV, respectively while all NS sequences from cattle (*n* = 19) originated from SisaV, and all NS of caprine (*n* = 15) belong to TusaV (Table 3). The acquired SisaV, MisaV and TusaV NS sequences show >83.2%, >92.7% and >84.5% pairwise nt identities, respectively.

Among different age groups, the highest prevalences of MisaV (91.7%) and SisaV (50.0%) were found in the second age group (2–12-month-old) followed by the third age group (>12 months) with a 41.7% and 25.0% MisaV and SisaV prevalence, while only two positive SisaV and no MisaV samples were found in the first age group (Table 3). Interestingly, besides the highest prevalence (100%) in the second age group, TusaVs were also detectable in the first and third age groups of caprine but with considerably lower prevalences (Table 3). TusaVs were also detectable in the first age group of ovine, like those found in our previous study [29] (Table 3).

**Figure 5:**
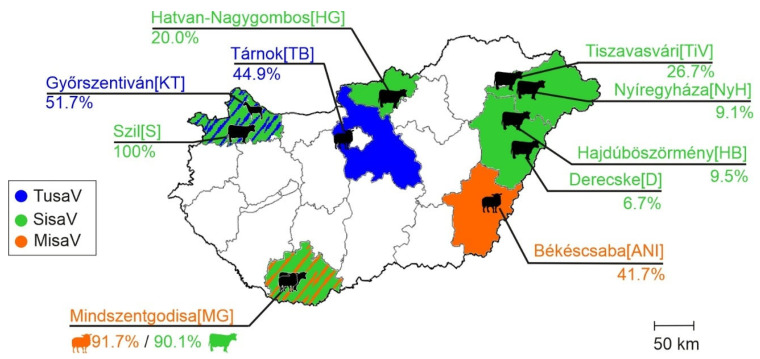
Geographical locations of misavirus (MisaV, orange), sisavirus (SisaV, green) and tusavirus (TusaV, blue) positive farms with overall prevalences. Counties with positive farms were also color-coded according to the viruses (see legend on the figure). Note that dual color patterns (e.g., blue-green or orange-green) indicate the presence of two viruses in that county.

MisaV was detected in two ovine farms (farm IDs: MG and ANI), and a total of seven bovine farms were SisaV-positive, while TusaVs were detectable only in an ovine (TB) and a caprine (KT) farm (Table 3 and Table S1, Figure 5).

**Figure 6:**
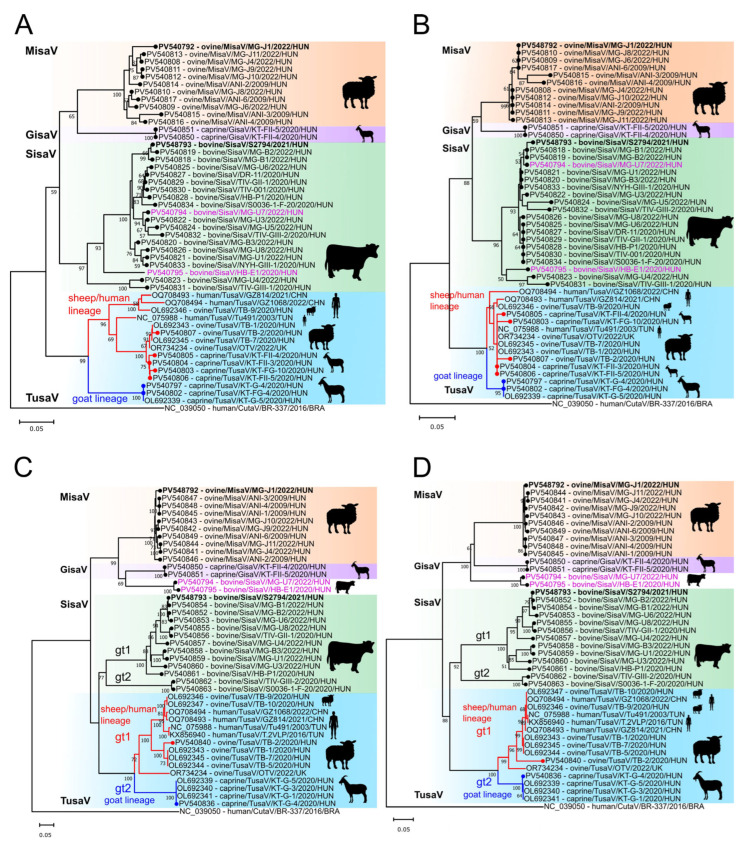
Phylogenetic analyses of the partial nucleotide (nt) and amino acid (aa) sequences of NS (NS-nt: A; NS-aa: B) and VP (VP-nt: C; VP-aa: D) of the study’s misa-, (MisaV, orange background), sisa-, (SisaV, green background) and tusaviruses (TusaV, blue background) including TusaVs identified by BLASTn searches. A human cutavirus sequence was used as an outgroup. The trees were generated by the Neighbor-Joining method with Jukes–Cantor (**A**,**C**) or p-distance (**B**,**D**) models with 1000 bootstrap (BS) replicates in MEGA ver11. BS values lower than 50 were removed from the tree. The index SisaV and MisaV sequences are marked with **bold**. Atypical sisavirus strains are marked with pink letters. The subsequently identified caprine gisavirus (GisaV) strains are marked with a purple background. The sequences identified in this study are marked with a circle in front of the strain names. The hosts of different strains are marked with silhouettes. gt: genotype. Two main sub-clades (gt1/ovine/human lineage and gt2/goat lineage) of TusaV are marked with red and blue lines.

Phylogenetically all the SisaV, MisaV and TusaV NS sequences form different, virus-specific main lineages that are separated from each other, although certain strains are located on different sub-lineages both on the nt and aa trees (Figure 6A,B). Unlike TusaV, all the SisaV and MisaV NS sequences were identified from bovine and ovine hosts, respectively. In the case of TusaVs, besides the previously described [29] caprine tusaviruses of goat TusaV lineage (KT-G samples) from the second age group, a total of four additional, previously undetected NS sequences from *n* = 3 dams (third age group) and a caprine kid (first age group) were also identified, which are more closely related to Hungarian ovine tusaviruses of the ovine/human TusaV lineage with high nt identities (up to 99.1%) rather than caprine tusaviruses from the same farm (KT-G samples) with considerably lower identities (up to 85.7%) (Figure 6A,B).

To investigate the capsid-based diversities in identified MisaV, SisaV and TusaV strains, generic primer pairs targeting the VP region of the viruses were designed and used on the NS-positive samples in a nested PCR setup. A ≈930 nt/310 aa long VP region could be determined in 13/19 NS PCR-positive ovine, 11/15 caprine and 13/19 bovine samples.

The acquired VP sequences were located on three different, virus-specific main clusters, but there are some strains among all three viruses that were located on multiple, well-defined sub-lineages in both the nt and aa trees (Figure 6C,D).

**Figure 7:**
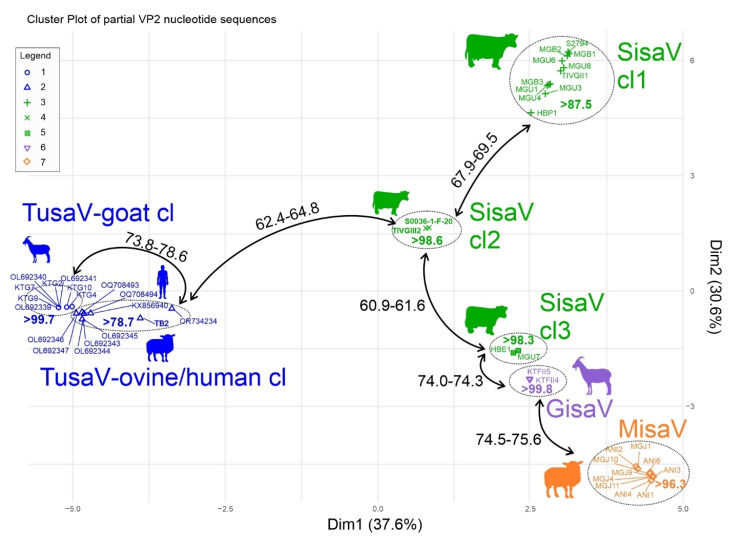
Hierarchical clustering analysis of the nucleotide alignments of the determined partial VP2 sequences (indicated with sample IDs), and the closely related tusavirus sequences (indicated with GenBank accession numbers). The hosts are illustrated with silhouettes. Sequences in the same cluster are marked with the same type of marker (see the legend on the figure) and circled with dotted lines. Clusters are color-coded according to the virus type (MisaV/misavirus, SisaV/sisavirus, GisaV/gisavirus, TusaV/tusavirus). cl: cluster. The lowest intracluster pairwise identity values (%) are marked inside the dotted circles/clusters. The highest ranges of intercluster identity values between sequences from different clusters are shown along the double arrows connecting those clusters. The representative ranges of intercluster identity values between sequences from different clusters are presented at the lines connecting different clusters.

Members of these sub-lineages could represent members of various tentative genotypes or species of these viruses. The sequences, which are phylogenetically related together in these separate sub-lineages, are also clustered together in the hierarchical clustering analysis. The clusters show considerably higher intracluster/intragenotype pairwise identity values, compared to the intercluster/intergenotype or interspecies values, except for ovine TusaV (OR734234), which also appeared distinct in the VP nt tree but not in the VP aa tree (Figure 7).

**Figure 8:**
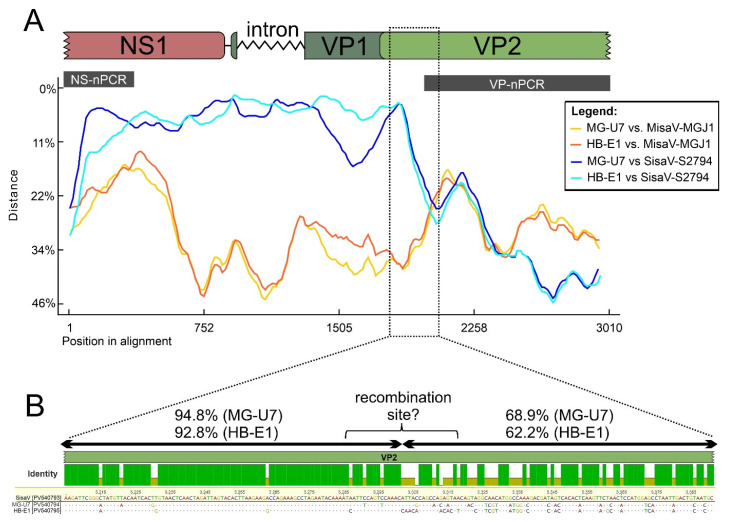
Merged results of distance plot analyses (**A**) of 2967 nt long genome regions of atypical sisavirus nucleotide sequences (MG-U7-PV540794; HB-E1-PV540795) compared with the corresponding genomic regions (see genomic map above the plot) of index misavirus (MisaV-MGJ1, PV540792, yellow and orange lines) and sisavirus (SisaV-S2794, PV540793, light and dark blue lines) strains. The positions of screening (NS-nPCR)/typing PCR products (VP-nPCR) are marked with gray bars above the plot. (**B**) Nucleotide alignment of partial VP2-encoding genome region including the potential recombination site of MG-U7, HB-E1 and the SisaV index sequence as a reference (PV540793). The location of the aligned region is marked with dotted lines on the schematic genome map. The identity graph above the alignment shows identical (green bars) and variable (pale yellow bars) nts of MG-U7 and HB-E1 compared to the SisaV index sequence. Only nts different from the SisaV index sequence are shown as letters in the alignments. Percentage values indicate the pairwise nt identities between SisaV index sequence and either MG-U7 or HB-E1 in the given region, indicated by double arrows.

While the phylogenetic positions of most MisaV, SisaV and TusaV strains in the VP trees correspond to the positions found in the NS trees, there are certain atypical SisaV sequences like MG-U7 (PV540794) and HB-E1 (PV540795) from bovine that are clustered together with the ovine MisaV strains instead of bovine SisaVs in both the nt and aa VP1 trees, although forming a well-defined sub-lineage (Figure 6C,D). Using NS- and VP-specific primers, PCR techniques and Sanger sequencing, continuous 2967 nt long genome regions of both strains were determined, spanning the 850nt/282 aa long partial NS and 1701nt/567 aa long partial VP1 (1245nt/415 aa long VP2) regions (Figure 8A). Distance plot analysis of the determined genome regions shows that both strains have a considerably lower (average of 7%) sequence distance to the SisaV reference sequence (S2794) compared to the MisaV reference (MG-J1, average of 36%) at the NS and 5′ VP2 regions, while the distance to SisaV increased considerably (from 7% to an average of 33%) but decreased to the MisaV reference (from 36% to an average of 29%) at the VP2 region (Figure 8A). The sharp decrease in identity between MG-U7/HB-E1 and SisaV-S2794 is clearly visible in the alignment of the corresponding 5′ VP2-encoding genome region, which could indicate a potential recombination site (Figure 8B). Recombination analyses did not reveal any recombination breakpoints due to the lack of closely related/parental sequence(s) at the VP2 region.

Interestingly, the acquired VP sequences from two of the caprine TusaV NS PCR-positive samples (KT-FII-4 and KT-FII-5) are clustered together with the MisaV-related VP sequences and form a distinct sub-lineage both in the nt and aa trees (Figure 6C,D). None of the PCR attempts were successful in connecting the TusaV-related NS and MisaV-related VP sequences, indicating the presence of two different protoparvoviruses in these two analyzed caprine samples.

### 3.3. Determination and Analyses of a Misavirus-Related Goat Protoparvoviruses (Gisaviruses)

**Figure 9:**
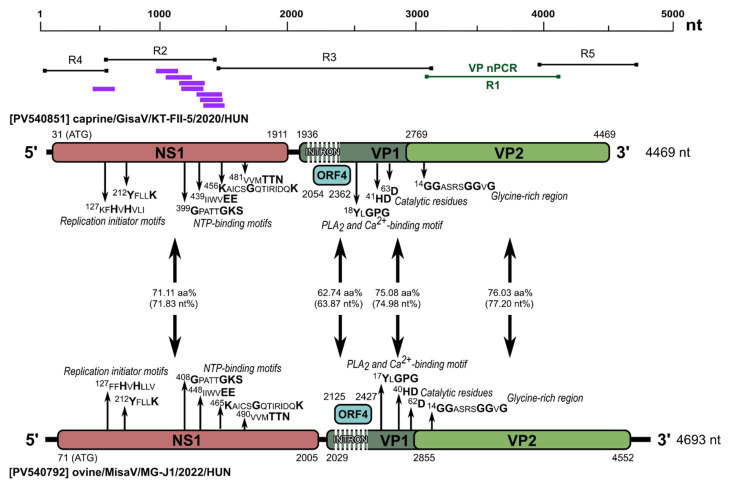
Schematic genome maps of caprine gisavirus (top) and ovine misavirus (bottom), including the characteristic parvoviral genome regions: ORF1 (NS1, pink), ORF2/3 (VP1/VP2, dark/light green) and ORF4 (hypothetical protein, light blue). Start and end nucleotide positions are shown above (gisavirus) and below (misavirus) the maps, along with NGS reads (purple), VP-typing PCRs (green) and genome-acquisition PCRs (black). Determined genome lengths are found on the right. Pairwise amino acid/aa (top) and nucleotide/nt (bottom, in brackets) identities (%) between regions are shown between boxes. Conserved parvoviral aa motifs are marked by the position of their first residue, with **bold** letters marking highly conserved residues.

To determine the complete coding sequences of misavirus-related goat protoparvoviruses identified by VP-nPCR, one of the positive caprine enteric samples (KT-FII-5) was subjected to VM-NGS. We identified *n* = 8 reads by analyzing the acquired NGS data as protoparvovirus sequences, which were then mapped to the index strain of ovine misavirus (MG-J1, PV540792) as the closest relative. Using read/VP sequence-specific and generic oligonucleotide primers targeting the conserved 3′ and 5′ genomic regions of misa- and sisavirus, the PCR method and Sanger sequencing techniques, a 4469 nt long CDS of caprine/GisaV/KT-FII-4/2020/HUN (PV570850) and caprine/GisaV/KT-FII-5/2020/HUN (PV570851) was determined from the two VP-nPCR positive caprine samples (KT-FII-4 and KT-FII-5), respectively. These caprine protoparvoviruses were tentatively named “gisaviruses” (GisaVs) based on the origins of the samples: Győrszentiván and stool-associated virus. The CDS of the two GisV strains share a 99.9% nt pairwise identity, and both show ~71.2% nt identities to the index ovine MisaV strain as their closest known relative. The coding genomes of both GisaV strains contain the same genome regions as MisaV, including the four ORFs (ORF1-4), a theoretical splicing donor/acceptor site of ORF2, and a potential donor site in ORF1 (Figure 9). The four ORFs of GisaV strains encode similar viral peptides (NS1 of ORF1, VP1/VP2 of ORF2/3, and a hypothetical protein encoded by ORF4) with similar conserved aa motifs as MisaV (Figure 9). The GisaV NS1 and VP1 contain an 18 aa long and 7 aa long deletion, while the hypothetical protein encoded by ORF4 contains a 2 aa long insertion compared to the corresponding proteins of MisaV, respectively. The pairwise nt/aa identities between ORFs/viral peptides of GisaV strain caprine/GisaV/KT-FII-5/2020/HUN and MisaV strain ovine/MisaV/MG-J1/HUN/2022 could be found in Figure 9. Phylogenetic analyses of the complete (Figures S1 and S2) and partial NS1 and VP proteins (Figure 6) indicate that GisaVs branch together with the misaviruses but form separate sub-lineages, and these viruses were also forming a separate cluster in the hierarchical clustering analysis as well (Figure 7).

## 4. Discussion

In this study, the complete coding sequences of three novel enteric protoparvoviruses, namely misavirus from ovine, sisavirus from bovine and subsequently gisavirus from caprine, were identified and genetically characterized in detail using viral metagenomics, next-generation sequencing and bioinformatics analyses. To our current knowledge sisavirus is the first published protoparvovirus from bovine species.

The two misavirus-related caprine gisaviruses were detected later after the genome characterizations of misa-, and sisavirus, serendipitously with generic primer pairs targeting the VP region but not with the NS primer pairs. The NS nPCR screening reactions detected only tusaviruses from these two samples, indicating the presence of TusaV/GisaV co-infections in these two asymptomatic dams. Retrospective analysis of NS screening primer target sites in GisaV sequences indicates up to four mismatches in both R1 and R2 reverse primers but not in the crucial first two residues of the 3′ region, suggesting that the NS primers could theoretically amplify GisaV as well, but in our cases, however, amplification predominantly favored TusaVs, likely due to their higher viral load. There were no additional GisaV-positive samples, which could be due to the high prevalence (up to 100% in age group II) of TusaVs in the investigated caprine samples of the given farm (farm KT) potentially obscuring the detection of co-infecting GisaVs. Further epidemiological studies are needed to assess the prevalence and genetic diversity of gisaviruses in caprine populations.

The genome organization of these three viruses follows the general protoparvovirus layout, including a potential intron region in the VP1 and with the presence of conserved protoparvoviral protein motifs in the NS and VP regions and a potential middle ORF (ORF4) which—unlike tusaviruses but like cuta- and bufaviruses—encodes a protein with a currently unknown function. The presence of a conserved potential splice donor site in ORF1 could indicate the existence of a spliced version of NS1 (probably NS2) in all the study viruses, similar to those found in other protoparvoviruses [55].

Based on the successful 5′RACE PCR reactions using both sense and antisense primer sets, the packaged virions of misa- and sisaviruses contain ssDNA genomes of both polarities with unknown proportions. No information was available about the polarity of the packaged genomes, especially among recently discovered protoparvoviruses like tusaviruses. To our current knowledge every parvoviral virion encapsidates one single positive or negative strand of ssDNA genome, adeno-associated virus 2 (AAV-2) of *genus Dependovirus* packages positive and negative strands equally, and members of the genus *Protoparvovirus* (e.g., *Protoparvovirus rodent 1*, MVM, *Protoparvovirus carnivoran 1*, CPV) predominantly encapsidate negative strands of genomes, while viruses in the genus *Bocaparvovirus*, like bovine parvovirus 1 (BPV-1), package both in different proportions [5,6,7].

The misa-, sisa- and gisaviruses display the highest sequence identities and closest phylogenetic relationship to ovine, caprine and human tusaviruses of species *Protoparvovirus incertum 1,* suggesting the common origin of these viruses.

Based on the results of phylogenetic analyses and sequence comparisons, as well as the species demarcation criteria of protoparvoviruses (a more than 85% aa identity in the replication initiator protein/NS1 for strains to be considered the same species [1]) misa-, sisa- and gisaviruses could be the founding members of three novel protoparvovirus species.

A small-scale epidemiological investigation was also conducted on multiple ruminant enteric samples, using novel generic nested primer pairs targeting the NS regions of known misa-, sisa- and tusaviruses. Our broad-range NS primers could not discriminate between the three viruses by PCR product size and therefore cannot be used to detect co-infections. Furthermore, in the case of multiple templates from different viruses in the sample/PCR product, the Sanger sequencing most likely reads only the dominant product, making minor templates undetectable [56]. The majority of the samples were positive only in the second (nested) PCR round, which could be due to the low viral load or the high genetic diversity of the parvoviral quasispecies present in the samples.

Both misa- and sisaviruses were identified by NS-based nested PCR in multiple ovine and bovine farms, respectively, which could indicate the endemic presence and continuous circulation of these viruses in the investigated farms.

Although the number of samples in different age groups is uneven, the considerably higher prevalences of misa- and sisaviruses in older (age groups II and III, 2–12-month-old, and >12-month-old) ovine and bovine animals compared to the younger animals is noticeable, which could indicate a later infection time or the prolonged incubation/shedding period of these protoparvoviruses. Meanwhile the DNA of the closest relative, tusavirus, was only detected in 5 out of a total of 5405 samples of human origin [22,23,24], so the prevalence in different age groups among humans could not be evaluated as yet; however, in small ruminants the highest prevalence was found among animals less than 1 year old [29,30]. In the case of cutavirus and bufaviruses in humans, the highest prevalence is found in populations older than 5 years old or adults [57,58].

Sequence and phylogenetic analyses of NS and VP sequences revealed the high genetic variability and loose phylogenetic relationship of the detected protoparvoviruses. In the case of tusaviruses, besides the positive ovine and caprine samples of age group II from farms TB and KT identified by our previous report [29], additional positive caprine samples from the same farm (KT) were found in animals of age groups I and III. These strains were undetectable with our previously used TusaV screening primers [29] due to mispriming, highlighting the need for the continuous re-design of tusavirus/protoparvovirus screening primers in animal and human molecular epidemiological studies. Interestingly, in the NS region these novel caprine TusaV strains are separated from the previously found caprine TusaV strains of the same farm [29] and clustered together with the ovine and human TusaV sequences, which could indicate the co-circulation of different TusaV strains (most likely different genotypes) in the investigated caprine farm. Unfortunately, despite several attempts with multiple TusaV primer pairs, including those which were used in our previous study [29], no VP sequences could be acquired from these novel caprine TusaV strains.

Partial VP-based phylogenetic analyses indicate the presence of multiple sub-lineages of both the nt and aa trees within misa-, and sisavirus, as well as tusavirus lineages which could correspond to different genotypes or even species. The presence of multiple genotypes among the study viruses (including tusaviruses) was also supported by a VP-based cluster analysis, where strains from the same phylogenetic sub-lineage were also clustered together. The rather low intra- and intercluster pairwise identity values could be due to the relatively high mutation rates of the study viruses, which are also a common feature of (proto)parvoviruses [8]. The measured partial VP capsid sequence-based genetic distances between clusters (potential genotypes) were similar in range with other protoparvoviruses, like bufaviruses, where the genotype criteria were tentatively set at <75% amino acid identities and supported by the phylogenetic analyses [14,16].

Interestingly, the presence of two bovine sisaviruses (MG-U7 and HB-E1) from two geographically distant cattle farms with diverse phylogenetic localizations in the NS (belonging to bovine sisaviruses) and VP trees (belonging to ovine misaviruses) could indicate a previous recombination event during the evolution of these strains. The parental sequences could be a sisavirus and a currently unknown misavirus genotype or more likely an unknown misavirus-related virus of a different species. The latter was supported by the marked phylogenetic separation of the VPs of MG-U7 and HB-E1 from misa- and gisaviruses. Based on the results of the distance plot analysis and sequence analysis of alignments of the capsid-encoding region, the recombination breakpoint could be somewhere at the 5′ part of the ORF2, but it cannot be identified more precisely due to the lack of a potential capsid–parental sequence(s). Recombination of (proto)parvoviruses is relatively common in closely related species, such as different genotypes of canine parvovirus 2, feline panleukopenia and mink enteritis viruses, even between the vaccine and wild strains, but interspecies recombinant protoparvoviruses are not described as yet [10,11,12,13].

Based on recent results (as well as the classification of TusaV as *Protoparvovirus incertum 1*), it was speculated that the original TusaV may not have been a human virus; rather, it may be of animal origin, which also points out the zoonotic potential of these viruses. Recently, two studies presented positive fecal samples for TusaV DNA from asymptomatic or diarrheic ovine and caprine in Hungary, as well as positive fecal and post-mortem tissue samples from lambs with lip lesions and pneumonia in the United Kingdom, with a high prevalence compared to human samples, which could indicate the ruminant origin of TusaVs [29,30]. The discovery of the three novel protoparvoviruses in this study from ruminants (ovine, bovine and caprine), which are located on the same main phylogenetic lineage as tusaviruses, as well as a novel potential genotype of tusaviruses from caprine, could further support the ruminant origin of tusaviruses, as well as misa-, sisa- and gisaviruses. However, it is necessary to investigate further the host spectrum of these parvoviruses.

The potential interspecies recombinant sisaviruses of bovine could suggest the capability to perform host species switches with unknown frequencies or even the zoonotic potential of these novel viruses, like those found in the closely related tusaviruses of the same main phylogenetic lineage. Additional epidemiological investigations are required to investigate the reservoirs and genetic/antigenic diversities, as well as the pathogenetic potential, of these ruminant protoparvoviruses, not just in animals but humans as well.

## Figures and Tables

**Table 1:** List of oligonucleotide primers used for epidemiologic investigation (NS screening) and typing (VP) in this study. VP: Capsid protein-, NS: non-structural protein-encoding genome region, nPCR: nested PCR. # The product sizes refer to misavirus strain ovine/MisaV/MG-J1/HUN/2022 (PV540792). *: PCR product sizes are the same in the tusa- and sisaviruses as well.

Target Region	Primer Name	5′-3′ Sequence	Reaction Type	Product Length (bp) #
NS	MiSiTuV-NS-Screen-F	TATGTRCACATIATGACTCA	Screening nPCR 1st PCR round	585 *
NS	MiSiTuV-NS-Screen-R	TCTGATYGGTTGAGTGTGTTC	Screening nPCR 1st PCR round	
NS	MiSiTuV-NS-Screen-F2	ACCTAGTTAARGAAAGACACAC	Screening nPCR 2nd PCR round	371 *
NS	MiSiTuV-NS-Screen-R2	TTGRTCTATGCGGATAGTTTG	Screening nPCR 2nd PCR round	
VP	MiSiTuV-VP-Fgen	CTGGTGAYTTTGACAATACTAC	Typing nPCR 1st PCR round	1285
VP	MiSiTuV-VP-Rgen	TTGTCCCAGATTTGTCCATGTGG	Typing nPCR 1st PCR round	
VP	MiSiTuV-VP-F2gen	AGCCTTGTAGACTGTAATGCATGG	Typing nPCR 2nd PCR round	1062
VP	MiSiTuV-VP-R2gen	TGGATAGTATGGACCTTGATGGTC	Typing nPCR 2nd PCR round	

**Table 2:** Sequence identity matrix (in percentages) based on the pairwise nucleotide (nt) and amino acid (aa) alignments of the signature viral proteins (NS1, VP1, VP2) of the study strains (misa- and sisavirus) and the most closely related human Tusavirus 1.

	Protein/Virus	Misavirus PV540792		Sisavirus PV540793	
Tusavirus 1 NC_075988		aa%	nt%	aa%	nt%
	NS1	63.04	67.02	62.14	67.61
	VP1	62.15	59.90	62.79	58.57
	VP2	62.12	67.66	63.89	67.07

**Table 3:** Age-related prevalence of misavirus (MisaV), sisavirus (SisaV) and tusavirus (TusaV) in ovine, bovine and caprine enteric samples from animals of three age groups (I, II, III) and from selected Hungarian farms. Prevalence data (in **bold**) were calculated after the results of sequence classifications of 328 nt long NS sequences from screening nested PCR reactions. ID: identification code of the farms, mo: months, n.a.: no available samples. Note that only farms with positive samples were listed. Locations of positive farms are found in Figure 5.

		No. of Positive Samples/Total by Age Groups								
		(I) <2 mo			(II) 2–12 mo			(III) >12 mo		
Host species	Farm location [ID]	MisaV	SisaV	TusaV	MisaV	SisaV	TusaV	MisaV	SisaV	TusaV
Ovine	Mindszentgodisa [MG]	n.a.	n.a.	n.a.	**11/12**	0/12	0/12	n.a.	n.a.	n.a.
	Tárnok [TB]	0/9	0/9	**4/9**	n.a.	n.a.	n.a.	n.a.	n.a.	n.a.
	Békéscsaba [ANI]	n.a.	n.a.	n.a.	n.a.	n.a.	n.a.	**5/12**	0/12	0/12
	**Ʃ**	0/9	0/9	**4/9** **(44.4%)**	**11/12** **(91.7%)**	0/12	0/12	**5/12** **(41.7%)**	0/12	0/12
Bovine	Hajdúböszörmény [HB]	0/19	0/19	0/19	n.a.	n.a.	n.a.	0/2	**2/2**	0/2
	Nyíregyháza [NyH]	0/5	0/5	0/5	0/4	0/4	0/4	0/2	**1/2**	0/2
	Szil [S]	n.a.	n.a.	n.a.	0/1	**1/1**	0/1	n.a.	n.a.	n.a.
	Derecske [DR]	0/3	0/3	0/3	0/1	0/1	0/1	0/11	**1/11**	0/11
	Tiszavasvári [TiV]	0/8	**1/8**	0/8	0/7	**3/7**	0/7	0/1	0/1	0/1
	Mindszentgodisa [MG]	n.a.	n.a.	n.a.	0/11	**10/11**	0/11	n.a.	n.a.	n.a.
	Hatvan-Nagygombos [HG]	0/5	**1/5**	0/5	n.a.	n.a.	n.a.	n.a.	n.a.	n.a.
	**Ʃ**	0/40	**2/40** **(5.0%)**	0/40	0/23	**14/23** **(60.86%)**	0/23	0/16	**4/16** **(25.0%)**	0/16
Caprine	Győrszentiván [KT]	0/9	0/9	**2/9**	0/10	0/10	**10/10**	0/10	0/10	**3/10**
	**Ʃ**	0/9	0/9	**2/9** **(22.2%)**	0/10	0/10	**10/10** **(100%)**	0/10	0/10	**3/10** **(30.0%)**

## Data Availability

The nucleotide sequence data reported in this study are available in the National Center for Biotechnology Information Database/GenBank database under the accession numbers PV540792-PV540863.

## Supplementary Materials

The following supporting information can be downloaded at: https://www.mdpi.com/article/10.3390/v17070888/s1, Figure S1: NS1 protein-based phylogenetic relationship of misa-, sisa-, and gisaviruses among members of the subfamily *Parvovirinae*, Figure S2: VP2 protein-based phylogenetic relationship of misa-, sisa-, and gisaviruses among members of genus *Protoparvovirus*; Table S1: Background information of individual samples used for the epidemiological investigation of misa-, sisa-, and tusaviruses.
