# Supplementary material for: Genomic Characterization and Molecular Epidemiology of Tusaviruses and Related Novel Protoparvoviruses (Family Parvoviridae) from Ruminant Species (Bovine, Ovine and Caprine) in Hungary
Source: Viruses. 2025 Jun 24;17(7):888. doi: 10.3390/v17070888 (PMC12300428; doi:10.3390/v17070888)
Supplement: Supplementary file 1 [file viruses-17-00888-s001.zip › Figure S1 - S2 -SUPPLEMENTARY.pdf]

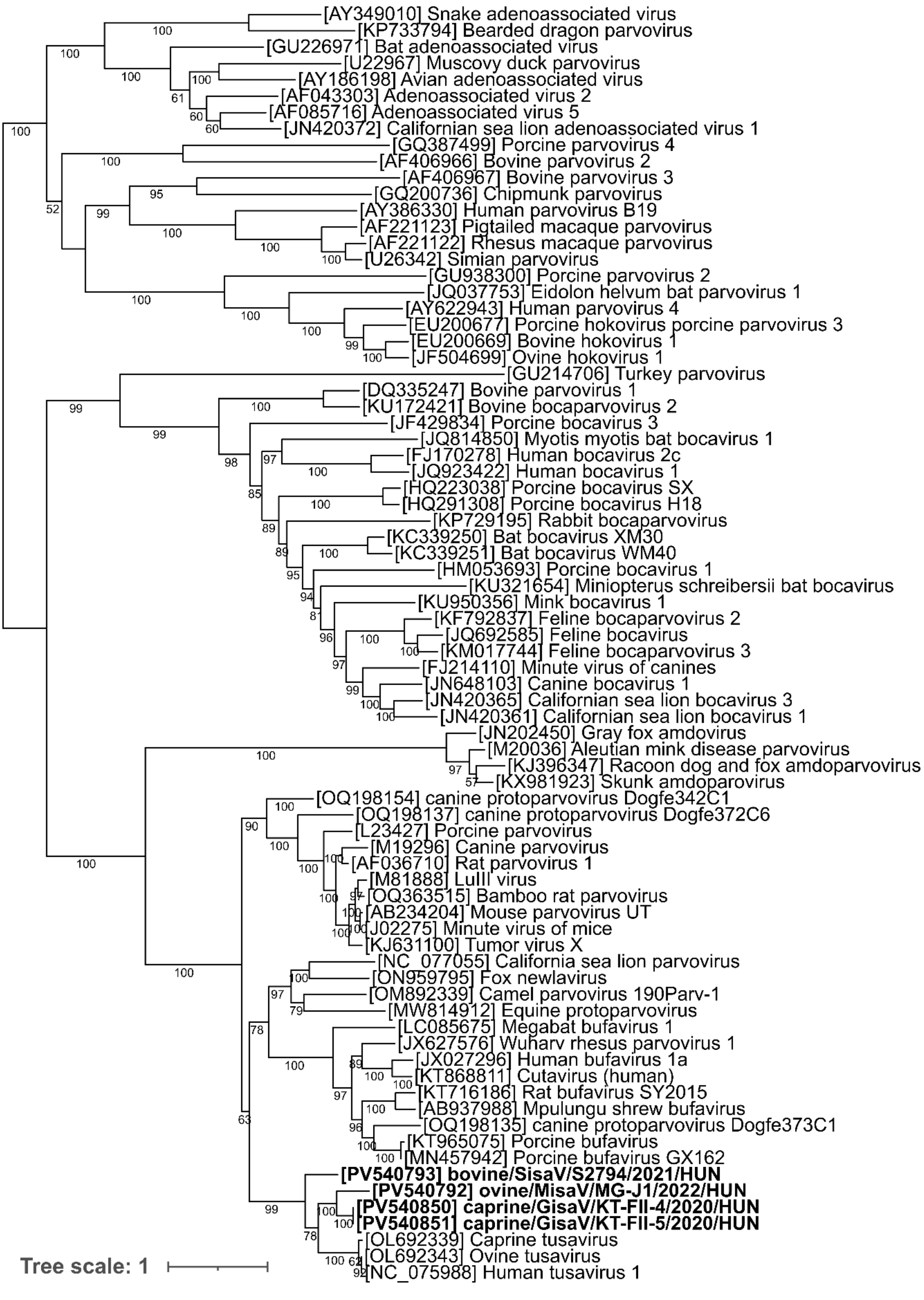

Figure S1.: Phylogenetic relationship of the study viruses (marked with **bold**), most closely related tusaviruses, and representative members of the subfamily *Parvovirinae*, based on the amino acid sequences of the full-length NS1 protein. The tree was generated by the Maximum likelihood method with an LG+F+I+G4 model with 1000 bootstrap (BS) replicates with IQTree, visualized with iTOL. Only BS values of 50 or more are indicated at the nodes.

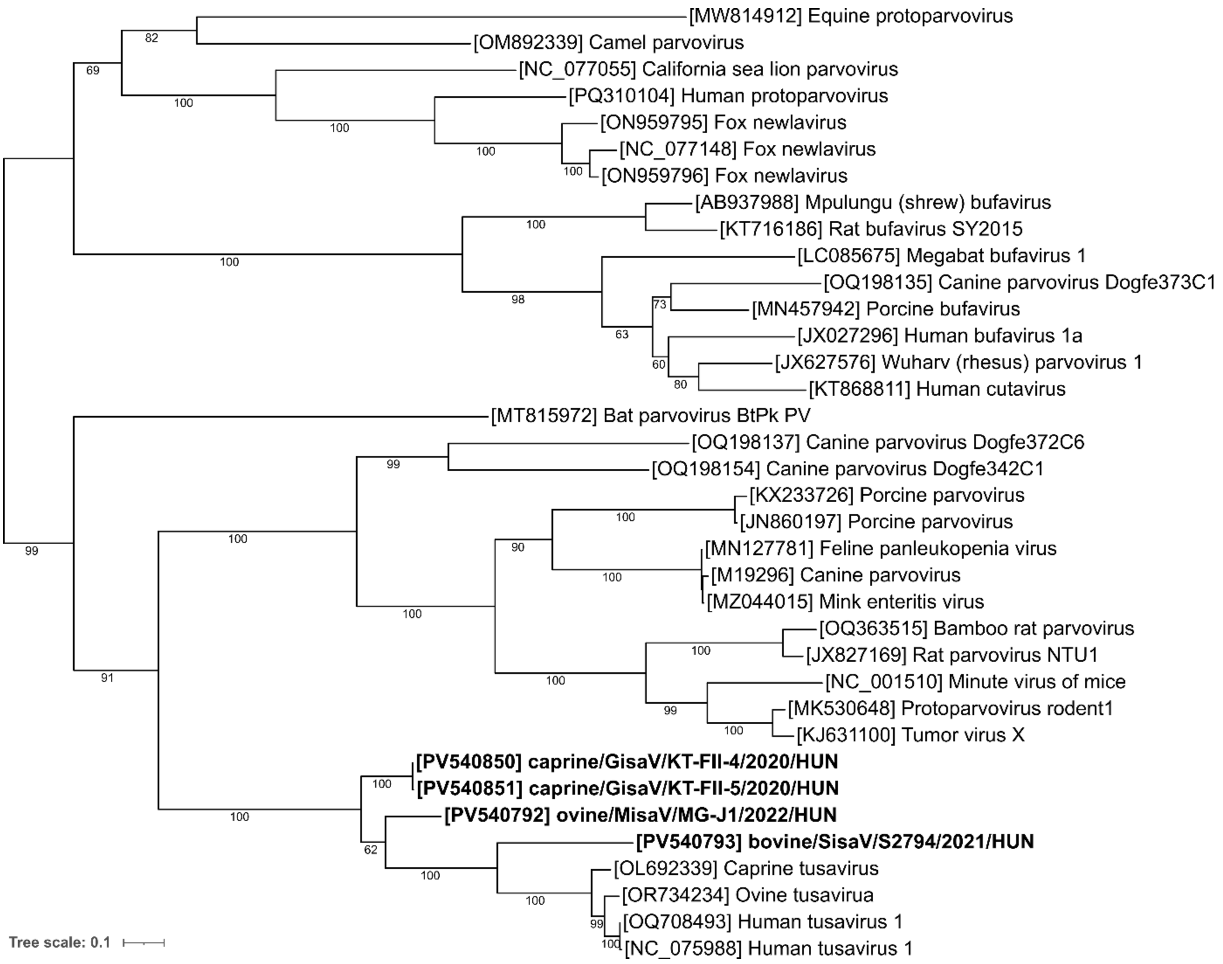

Figure S2.: Phylogenetic analysis of the study viruses (marked with **bold**), the most closely related tusaviruses and representative members of genus *Protoparvovirus*, based on the amino acid sequences of the full-length VP2 viral protein. The tree was generated by the Maximum likelihood method with an LG+F+I+G4 model with 1000 bootstrap (BS) replicates with IQTree, visualized with iTOL. Only BS values of 50 or more are indicated at the nodes.
