# Supplementary material for: Genomic Characterization and Molecular Epidemiology of Tusaviruses and Related Novel Protoparvoviruses (Family Parvoviridae) from Ruminant Species (Bovine, Ovine and Caprine) in Hungary
Source: Viruses. 2025 Jun 24;17(7):888. doi: 10.3390/v17070888 (PMC12300428; doi:10.3390/v17070888)
Supplement: Supplementary file 1 [file viruses-17-00888-s001.zip › TableS1-SUPPLEMENTARY.pdf]

Farm location	Sample ID	Host species	Age groups	MiSiTuV-NSP-Screen PCR1	MiSiTuV-NSP-Screen PCR2	MiSiTuV-VP-typing PCR2	Accession numbers
Hajdúböszörmény	HB-7369	bovine	I	negative	negative	n.p.	
Hajdúböszörmény	HB-7660	bovine	I	negative	negative	n.p.	
Hajdúböszörmény	HB-7373	bovine	I	negative	negative	n.p.	
Hajdúböszörmény	HB-7431	bovine	I	negative	negative	n.p.	
Hajdúböszörmény	HB-7396	bovine	I	negative	negative	n.p.	
Hajdúböszörmény	HB-7693	bovine	I	negative	negative	n.p.	
Hajdúböszörmény	HB-7433	bovine	I	negative	negative	n.p.	
Hajdúböszörmény	HB-7248	bovine	I	negative	negative	n.p.	
Hajdúböszörmény	HB-7615	bovine	I	negative	negative	n.p.	
Hajdúböszörmény	HB-7315	bovine	I	negative	negative	n.p.	
Hajdúböszörmény	HB-7657	bovine	I	negative	negative	n.p.	
Hajdúböszörmény	HB-7500	bovine	I	negative	negative	n.p.	
Hajdúböszörmény	HB-7673	bovine	I	negative	negative	n.p.	
Hajdúböszörmény	HB-7627	bovine	I	negative	negative	n.p.	
Hajdúböszörmény	HB-7751	bovine	I	negative	negative	n.p.	
Hajdúböszörmény	HB-8066	bovine	I	negative	negative	n.p.	
Hajdúböszörmény	HB-0303	bovine	I	negative	negative	n.p.	
Hajdúböszörmény	HB-7663	bovine	I	negative	negative	n.p.	
Hajdúböszörmény	HB-7394	bovine	I	negative	negative	n.p.	
Hajdúböszörmény	HB-E-1	bovine	III	negative	positive	positive	NSP&VP: PV540795
Hajdúböszörmény	HB-P1	bovine	III	negative	positive	positive	NSP: PV540828; VP: PV540861
Nyíregyháza	NYH-3175	bovine	I	negative	negative	n.p.	
Nyíregyháza	NYH-3254	bovine	I	negative	negative	n.p.	
Nyíregyháza	NYH-3257	bovine	I	negative	negative	n.p.	
Nyíregyháza	NYH-3259	bovine	I	negative	negative	n.p.	
Nyíregyháza	NYH-3256	bovine	I	negative	negative	n.p.	
Nyíregyháza	NYH-GI-1	bovine	II	negative	negative	n.p.	
Nyíregyháza	NYH-GI-2	bovine	II	negative	negative	n.p.	
Nyíregyháza	NYH-GII-1	bovine	II	negative	negative	n.p.	
Nyíregyháza	NYH-GII-2	bovine	II	negative	negative	n.p.	
Nyíregyháza	NYH-GIII-1	bovine	III	negative	positive	negative	PV540833
Nyíregyháza	NYH-GIII-2	bovine	III	negative	negative	n.p.	
Derecske	DR-B-1	bovine	I	negative	negative	n.p.	
Derecske	DR-B-2	bovine	I	negative	negative	n.p.	
Derecske	DR-B-3	bovine	I	negative	negative	n.p.	
Derecske	DR-B-4	bovine	II	negative	negative	n.p.	
Derecske	DR-1	bovine	III	negative	negative	n.p.	
Derecske	DR-2	bovine	III	negative	negative	n.p.	
Derecske	DR-3	bovine	III	negative	negative	n.p.	
Derecske	DR-4	bovine	III	negative	negative	n.p.	
Derecske	DR-5	bovine	III	negative	negative	n.p.	
Derecske	DR-6	bovine	III	negative	negative	n.p.	

Derecske	DR-7	bovine	III	negative	negative	n.p.	
Derecske	DR-8	bovine	III	negative	negative	n.p.	
Derecske	DR-9	bovine	III	negative	negative	n.p.	
Derecske	DR-10	bovine	III	negative	negative	n.p.	
Derecske	DR-11	bovine	III	negative	positive	negative	PV540827
Szil	**S2794**	bovine	I	positive	positive	positive	**PV540793#**
Sárospatak	0010-1-F/20	bovine	I	negative	negative	n.p.	
Sárospatak	0010-2-F/20	bovine	I	negative	negative	n.p.	
Sárospatak	0010-4-F/20	bovine	I	negative	negative	n.p.	
Sárospatak	0010-3-F/20	bovine	I	negative	negative	n.p.	
Sárospatak	0010-5-F/20	bovine	I	negative	negative	n.p.	
Kenézlő	0011-1-F/20	bovine	I	negative	negative	n.p.	
Kenézlő	0011-2-F/20	bovine	I	negative	negative	n.p.	
Kenézlő	0011-3-F/20	bovine	I	negative	negative	n.p.	
Kenézlő	0011-4-F/20	bovine	I	negative	negative	n.p.	
Kenézlő	0011-5-F/20	bovine	I	negative	negative	n.p.	
Hatvan-Nagygombos	0036-1-F/20	bovine	I	negative	positive	positive	NSP: PV540834; VP: PV540863
Hatvan-Nagygombos	0036-2-F/20	bovine	I	negative	negative	n.p.	
Hatvan-Nagygombos	0036-3-F/20	bovine	I	negative	negative	n.p.	
Hatvan-Nagygombos	0036-4-F/20	bovine	I	negative	negative	n.p.	
Hatvan-Nagygombos	0036-5-F/20	bovine	I	negative	negative	n.p.	
Törtel	0045-1-F/20	bovine	I	negative	negative	n.p.	
Törtel	0045-2-F/20	bovine	I	negative	negative	n.p.	
Törtel	0045-3-F/20	bovine	I	negative	negative	n.p.	
Törtel	0045-4-F/20	bovine	I	negative	negative	n.p.	
Törtel	0045-5-F/20	bovine	I	negative	negative	n.p.	
Pély	0050-1-F/20	bovine	I	negative	negative	n.p.	
Pély	0050-2-F/20	bovine	I	negative	negative	n.p.	
Pély	0050-3-F/20	bovine	I	negative	negative	n.p.	
Pély	0050-4-F/20	bovine	I	negative	negative	n.p.	
Pély	0050-5-F/20	bovine	I	negative	negative	n.p.	
Pély	0095-1-F/20	bovine	I	negative	negative	n.p.	
Pély	0095-4-F/20	bovine	I	negative	negative	n.p.	
Hajdúböszörmény	0066-1-F/20	bovine	I	negative	negative	n.p.	
Hajdúböszörmény	0066-2-F/20	bovine	I	negative	negative	n.p.	
Hajdúböszörmény	0066-3-F/20	bovine	I	negative	negative	n.p.	
Hajdúböszörmény	0066-4-F/20	bovine	I	negative	negative	n.p.	
Hajdúböszörmény	0066-5-F/20	bovine	I	negative	negative	n.p.	
Hajdúböszörmény	0117-2-F/20	bovine	I	negative	negative	n.p.	
Hajdúnánás	0067-1-F/20	bovine	I	negative	negative	n.p.	
Hajdúnánás	0067-2-F/20	bovine	I	negative	negative	n.p.	
Hajdúnánás	0067-3-F/20	bovine	I	negative	negative	n.p.	
Hajdúnánás	0067-4-F/20	bovine	I	negative	negative	n.p.	

Hajdúnánás	0067-5-F/20	bovine	I	negative	negative	n.p.	
Derecske	0092-1-F/20	bovine	I	negative	negative	n.p.	
Derecske	0092-2-F/20	bovine	I	negative	negative	n.p.	
Derecske	0092-3-F/20	bovine	I	negative	negative	n.p.	
Derecske	0092-4-F/20	bovine	I	negative	negative	n.p.	
Derecske	0092-6-F/20	bovine	I	negative	negative	n.p.	
Derecske	0093-1-F/20	bovine	I	negative	negative	n.p.	
Derecske	0093-2-F/20	bovine	I	negative	negative	n.p.	
Bonyhád	0121-1-F/20	bovine	I	negative	negative	n.p.	
Tiszavasvári	1412-1-F/19	bovine	I	negative	negative	n.p.	
Tiszavasvári	1412-2-F/19	bovine	I	negative	negative	n.p.	
Tiszavasvári	1412-3-F/19	bovine	I	negative	negative	n.p.	
Tiszavasvári	1412-4-F/19	bovine	I	negative	negative	n.p.	
Tiszavasvári	1412-5-F/19	bovine	I	negative	negative	n.p.	
Kazsok	0089-1-F/20	bovine	I	negative	negative	n.p.	
Kazsok	0089-2-F/20	bovine	I	negative	negative	n.p.	
Kazsok	0089-3-F/20	bovine	I	negative	negative	n.p.	
Kazsok	1441-10-F-19	bovine	I	negative	negative	n.p.	
Kazsok	1441-5-F/19	bovine	I	negative	negative	n.p.	
Kazsok	1441-6-F/19	bovine	I	negative	negative	n.p.	
Kazsok	1441-7-F/19	bovine	I	negative	negative	n.p.	
Kazsok	1441-19/1-F	bovine	I	negative	negative	n.p.	
Kazsok	1441-3-F/19	bovine	I	negative	negative	n.p.	
Kazsok	1441-4-F/19	bovine	I	negative	negative	n.p.	
Hajdúböszörmény	1442-19-1-F	bovine	I	negative	negative	n.p.	
Hajdúböszörmény	1442-19-2-F	bovine	I	negative	negative	n.p.	
Hajdúböszörmény	1442-19-3-F	bovine	I	negative	negative	n.p.	
Hajdúböszörmény	1442-19-4-F	bovine	I	negative	negative	n.p.	
Hajdúböszörmény	1442-19-5-F	bovine	I	negative	negative	n.p.	
Igrici	1481-1-F-19	bovine	I	negative	negative	n.p.	
Igrici	1481-2-F/19	bovine	I	negative	negative	n.p.	
Igrici	1481-3-F/19	bovine	I	negative	negative	n.p.	
Igrici	1481-5-F/19	bovine	I	negative	negative	n.p.	
Igrici	1481-4-F/19	bovine	I	negative	negative	n.p.	
Igrici	1447-19/1-F	bovine	I	negative	negative	n.p.	
Igrici	1447-19/3-F	bovine	I	negative	negative	n.p.	
Igrici	1447-19/4-F	bovine	I	negative	negative	n.p.	
Igrici	1447-19/5-F	bovine	I	negative	negative	n.p.	
Komárom	1316-19-1-F	bovine	I	negative	negative	n.p.	
Komárom	1316-2-F-19	bovine	I	negative	negative	n.p.	
Komárom	1316-4-F-19	bovine	I	negative	negative	n.p.	
Komárom	1316-3-F-19	bovine	I	negative	negative	n.p.	
Komárom	1316-5-F-19	bovine	I	negative	negative	n.p.	
Komárom	1426-19/1-F	bovine	I	negative	negative	n.p.	

Komárom	1426-2-F/19	bovine	I	negative	negative	n.p.	
Komárom	1426-4-F/19	bovine	I	negative	negative	n.p.	
Komárom	1426-3-F/19	bovine	I	negative	negative	n.p.	
Komárom	1426-5-F/19	bovine	I	negative	negative	n.p.	
Szombathely	0033-1-F/20	bovine	I	negative	negative	n.p.	
Szombathely	0033-2-F/20	bovine	I	negative	negative	n.p.	
Szombathely	0033-3-F/20	bovine	I	negative	negative	n.p.	
Szombathely	0033-4-F/20	bovine	I	negative	negative	n.p.	
Szombathely	0065-3-F/20	bovine	I	negative	negative	n.p.	
Szombathely	0065-4-F/20	bovine	I	negative	negative	n.p.	
Szombathely	0065-5-F/20	bovine	I	negative	negative	n.p.	
Szombathely	0090-1-F/20	bovine	I	negative	negative	n.p.	
Szombathely	0090-2-F/20	bovine	I	negative	negative	n.p.	
Szombathely	0090-3-F/20	bovine	I	negative	negative	n.p.	
Szombathely	1478-2-F/19	bovine	I	negative	negative	n.p.	
Tiszavasvári	TiV-001	bovine	I	negative	positive	negative	PV540830
Tiszavasvári	TiV-002	bovine	I	negative	negative	n.p.	
Tiszavasvári	TiV-003	bovine	I	negative	negative	n.p.	
Tiszavasvári	TiV-004	bovine	I	negative	negative	n.p.	
Tiszavasvári	TiV-005	bovine	I	negative	negative	n.p.	
Tiszavasvári	TiV-007	bovine	I	negative	negative	n.p.	
Tiszavasvári	TiV-6379	bovine	I	negative	negative	n.p.	
Tiszavasvári	TiV-0577	bovine	I	negative	negative	n.p.	
Tiszavasvári	TiV-GI-1	bovine	II	negative	negative	n.p.	
Tiszavasvári	TiV-GI-2	bovine	II	negative	negative	n.p.	
Tiszavasvári	TiV-GI-3	bovine	II	negative	negative	n.p.	
Tiszavasvári	TiV-GII-1	bovine	II	positive	positive	positive	NSP: PV540829;<br>VP: PV540856
Tiszavasvári	TiV-GII-2	bovine	II	negative	negative	n.p.	
Tiszavasvári	TiV-GIII-1	bovine	II	negative	positive	negative	PV540831
Tiszavasvári	TiV-GIII-2	bovine	II	negative	positive	positive	NSP: PV540832;<br>VP: PV540862
Tiszavasvári	TiV-F1	bovine	III	negative	negative	n.p.	
Bonyhád	BH-0001	bovine	I	negative	negative	n.p.	
Bonyhád	BH-9812	bovine	I	negative	negative	n.p.	
Bonyhád	BH-9852	bovine	I	negative	negative	n.p.	
Bonyhád	BH-9854	bovine	I	negative	negative	n.p.	
Bonyhád	BH-9858	bovine	I	negative	negative	n.p.	
Bonyhád	BH-9861	bovine	I	negative	negative	n.p.	
Bonyhád	BH-9865	bovine	I	negative	negative	n.p.	
Bonyhád	BH-9878	bovine	I	negative	negative	n.p.	
Bonyhád	BH-9901	bovine	I	negative	negative	n.p.	
Bonyhád	BH-9955	bovine	I	negative	negative	n.p.	
Bonyhád	BH-9963	bovine	I	negative	negative	n.p.	

Bonyhád	BH-9971	bovine	I	negative	negative	n.p.	
Bonyhád	BH-9974	bovine	I	negative	negative	n.p.	
Bonyhád	BH-9989	bovine	I	negative	negative	n.p.	
Bonyhád	BH-9993	bovine	I	negative	negative	n.p.	
Bonyhád	BH-9994	bovine	I	negative	negative	n.p.	
Tevel	TV-9682	bovine	I	negative	negative	n.p.	
Tevel	TV-9686	bovine	I	negative	negative	n.p.	
Tevel	TV-9703	bovine	I	negative	negative	n.p.	
Tevel	TV-9728	bovine	I	negative	negative	n.p.	
Tevel	TV-9733	bovine	I	negative	negative	n.p.	
Tevel	TV-9736	bovine	I	negative	negative	n.p.	
Tevel	TV-9738	bovine	I	negative	negative	n.p.	
Tevel	TV-9743	bovine	I	negative	negative	n.p.	
Tevel	TV-9746	bovine	I	negative	negative	n.p.	
Tevel	TV-9760	bovine	I	negative	negative	n.p.	
Tevel	TV-9764	bovine	I	negative	negative	n.p.	
Tevel	TV-9772	bovine	I	negative	negative	n.p.	
Tevel	TV-9774	bovine	I	negative	negative	n.p.	
Tevel	TV-9779	bovine	I	negative	negative	n.p.	
Tevel	TV-9785	bovine	I	negative	negative	n.p.	
Tevel	TV-9787	bovine	I	negative	negative	n.p.	
Tevel	TV-9789	bovine	I	negative	negative	n.p.	
Mindszentgodisa	MG U 1	bovine	II	negative	positive	positive	NSP: PV540821; VP: PV540859
Mindszentgodisa	MG U 2	bovine	II	negative	negative	n.p.	
Mindszentgodisa	MG U 3	bovine	II	negative	positive	positive	NSP: PV540822; VP: PV540860
Mindszentgodisa	MG U 4	bovine	II	negative	positive	negative	PV540823
Mindszentgodisa	MG U 5	bovine	II	negative	positive	negative	PV540824
Mindszentgodisa	MG U 6	bovine	II	negative	positive	positive	NSP: PV540825; VP: PV540853
Mindszentgodisa	MG U 7	bovine	II	positive	positive	positive	NSP&VP: PV540794
Mindszentgodisa	MG U 8	bovine	II	negative	positive	positive	NSP: PV540826; VP: PV540855
Mindszentgodisa	MG B 1	bovine	II	negative	positive	positive	NSP: PV540818; VP: PV540854
Mindszentgodisa	MG B 2	bovine	II	negative	positive	positive	NSP: PV540819; VP: PV540852
Mindszentgodisa	MG B 3	bovine	II	negative	positive	positive	NSP: PV540820; VP: PV540858
Mindszentgodisa	MG J 1	ovine	II	positive	positive	positive	**PV540792#**
Mindszentgodisa	MG J 2	ovine	II	positive	positive	negative	
Mindszentgodisa	MG J 3	ovine	II	positive	positive	negative	

Mindszentgodisa	MG J 4	ovine	II	positive	positive	positive	NSP: PV540808;<br>VP: PV540841
Mindszentgodisa	MG J 5	ovine	II	positive	positive	negative	
Mindszentgodisa	MG J 6	ovine	II	negative	positive	negative	PV540809
Mindszentgodisa	MG J 7	ovine	II	positive	positive	negative	
Mindszentgodisa	MG J 8	ovine	II	positive	positive	negative	PV540810
Mindszentgodisa	MG J 9	ovine	II	positive	positive	positive	NSP: PV540811;<br>VP: PV540842
Mindszentgodisa	MG J 10	ovine	II	negative	positive	positive	NSP: PV540812;<br>VP: PV540843
Mindszentgodisa	MG J 11	ovine	II	positive	positive	positive	NSP: PV540813;<br>VP: PV540844
Mindszentgodisa	MG J 12	ovine	II	negative	negative	n.p.	
Hajdúszoboszló	HBSZ-B-GII-1	ovine	I	negative	negative	n.p.	
Hajdúszoboszló	HBSZ-B-GII-2	ovine	I	negative	negative	n.p.	
Hajdúszoboszló	HBSZ-B-GII-3	ovine	I	negative	negative	n.p.	
Hajdúszoboszló	HBSZ-B-GII-4	ovine	I	negative	negative	n.p.	
Hajdúszoboszló	HBSZ-B-GII-5	ovine	I	negative	negative	n.p.	
Hajdúszoboszló	HBSZ-B-GIII-1	ovine	I	negative	negative	n.p.	
Hajdúszoboszló	HBSZ-B-GIII-2	ovine	I	negative	negative	n.p.	
Hajdúszoboszló	HBSZ-B-GIII-3	ovine	I	negative	negative	n.p.	
Hajdúszoboszló	HBSZ-B-GI-1	ovine	II	negative	negative	n.p.	
Hajdúszoboszló	HBSZ-B-GI-2	ovine	II	negative	negative	n.p.	
Hajdúszoboszló	HBSZ-B-GI-3	ovine	II	negative	negative	n.p.	
Hajdúszoboszló	HBSZ-B-GI-4	ovine	II	negative	negative	n.p.	
Hajdúszoboszló	HBSZ-B-GI-5	ovine	II	negative	negative	n.p.	
Hajdúszoboszló	HBSZ-B-GIV-1	ovine	III	negative	negative	n.p.	
Hajdúszoboszló	HBSZ-B-GIV-2	ovine	III	negative	negative	n.p.	
Hajdúszoboszló	HBSZ-B-GV-1	ovine	III	negative	negative	n.p.	
Hajdúszoboszló	HBSZ-B-GV-2	ovine	III	negative	negative	n.p.	
Hajdúszoboszló	HBSZ-K-1	ovine	III	negative	negative	n.p.	
Hajdúszoboszló	HBSZ-TL-1	ovine	III	negative	negative	n.p.	
Tárnok	TB-1	ovine	I	negative	positive	positive	Identical to: OL692343 [29]
Tárnok	TB-2	ovine	I	negative	positive	positive	NSP: PV540807;<br>VP: PV540840
Tárnok	TB-3	ovine	I	negative	negative	n.p.	
Tárnok	TB-4	ovine	I	negative	negative	n.p.	
Tárnok	TB-5	ovine	I	negative	negative	n.p.	
Tárnok	TB-6	ovine	I	negative	negative	n.p.	
Tárnok	TB-7	ovine	I	negative	positive	positive	Identical to: OL692345 [29]
Tárnok	TB-8	ovine	I	negative	negative	n.p.	

Tárnok	TB-9	ovine	I	positive	positive	positive	Identical to: OL692346 [29]
Békéscsaba	ANI-1	ovine	III	negative	positive	positive	VP: PV540845
Békéscsaba	ANI-2	ovine	III	negative	positive	positive	NSP: PV540814; VP: PV540846
Békéscsaba	ANI-3	ovine	III	negative	positive	positive	NSP: PV540815; VP: PV540847
Békéscsaba	ANI-4	ovine	III	negative	positive	positive	NSP: PV540816; VP: PV540848
Békéscsaba	ANI-5	ovine	III	negative	negative	n.p.	
Békéscsaba	ANI-6	ovine	III	negative	positive	positive	NSP: PV540817; VP: PV540849
Békéscsaba	ANI-7	ovine	III	negative	negative	n.p.	
Békéscsaba	ANI-8	ovine	III	negative	negative	n.p.	
Békéscsaba	ANI-9	ovine	III	negative	negative	n.p.	
Békéscsaba	ANI-10	ovine	III	negative	negative	n.p.	
Békéscsaba	ANI-11	ovine	III	negative	negative	n.p.	
Békéscsaba	ANI-12	ovine	III	negative	negative	n.p.	
Aranyosgadány	AGK-9	caprine	II	negative	negative	n.p.	
Aranyosgadány	AGK-10	caprine	II	negative	negative	n.p.	
Aranyosgadány	AGK-11	caprine	II	negative	negative	n.p.	
Aranyosgadány	AGK-12	caprine	II	negative	negative	n.p.	
Aranyosgadány	AGK-13	caprine	II	negative	negative	n.p.	
Aranyosgadány	AGK-14	caprine	II	negative	negative	n.p.	
Aranyosgadány	AGK-15	caprine	II	negative	negative	n.p.	
Aranyosgadány	AGK-16	caprine	II	negative	negative	n.p.	
Aranyosgadány	AGK-1	caprine	III	negative	negative	n.p.	
Aranyosgadány	AGK-2	caprine	III	negative	negative	n.p.	
Aranyosgadány	AGK-3	caprine	III	negative	negative	n.p.	
Aranyosgadány	AGK-4	caprine	III	negative	negative	n.p.	
Aranyosgadány	AGK-5	caprine	III	negative	negative	n.p.	
Aranyosgadány	AGK-6	caprine	III	negative	negative	n.p.	
Aranyosgadány	AGK-7	caprine	III	negative	negative	n.p.	
Aranyosgadány	AGK-8	caprine	III	negative	negative	n.p.	
Győrszentiván	KT-FG-2	caprine	I	negative	negative	n.p.	
Győrszentiván	KT-FG-3	caprine	I	negative	negative	n.p.	
Győrszentiván	KT-FG-4	caprine	I	negative	positive	negative	PV540802
Győrszentiván	KT-FG-5	caprine	I	negative	negative	n.p.	
Győrszentiván	KT-FG-6	caprine	I	negative	negative	n.p.	
Győrszentiván	KT-FG-7	caprine	I	negative	negative	n.p.	
Győrszentiván	KT-FG-8	caprine	I	negative	negative	n.p.	
Győrszentiván	KT-FG-9	caprine	I	negative	negative	n.p.	
Győrszentiván	KT-FG-10	caprine	I	negative	positive	positive	NSP: PV540803 VP: PV540839

Győrszentiván	KT-G-1	caprine	II	negative	positive	positive	Identical to: OL692341 [29]
Győrszentiván	KT-G-2	caprine	II	positive	positive	positive	NSP: PV540796; VP: PV540835
Győrszentiván	KT-G-3	caprine	II	positive	positive	positive	Identical to: OL692340 [29]
Győrszentiván	KT-G-4	caprine	II	negative	positive	positive	NSP: PV540797; VP: PV540836
Győrszentiván	KT-G-5	caprine	II	positive	positive	positive	Identical to: OL692339 [29]
Győrszentiván	KT-G-6	caprine	II	negative	positive	negative	
Győrszentiván	KT-G-7	caprine	II	positive	positive	positive	NSP: PV540798; VP: PV540837
Győrszentiván	KT-G-8	caprine	II	positive	positive	positive	NSP: PV540799
Győrszentiván	KT-G-9	caprine	II	positive	positive	positive	NSP: PV540800; VP: PV540838
Győrszentiván	KT-G-10	caprine	II	negative	positive	negative	PV540801
Győrszentiván	KT-FI-1	caprine	III	negative	negative	n.p.	
Győrszentiván	KT-FI-2	caprine	III	negative	negative	n.p.	
Győrszentiván	KT-FI-3	caprine	III	negative	negative	n.p.	
Győrszentiván	KT-FI-4	caprine	III	negative	negative	n.p.	
Győrszentiván	KT-FI-5	caprine	III	negative	negative	n.p.	
Győrszentiván	KT-FII-1	caprine	III	negative	negative	n.p.	
Győrszentiván	KT-FII-2	caprine	III	negative	negative	n.p.	
Győrszentiván	KT-FII-3	caprine	III	negative	positive	negative	PV540804
Győrszentiván	KT-FII-4	caprine	III	negative	positive	positive	NSP: PV540805 (TusaV); VP/CDS: **PV540850#** (GisaV)
Győrszentiván	KT-FII-5	caprine	III	negative	positive	positive	NSP: PV540806 (TusaV); VP/CDS: **PV540851#** (GisaV)
Nagyhegy	NH-1	caprine	III	negative	negative	n.p.	
Nagyhegy	NH-2	caprine	III	negative	negative	n.p.	
Nagyhegy	NH-3	caprine	III	negative	negative	n.p.	
Nagyhegy	NH-4	caprine	III	negative	negative	n.p.	
Nagyhegy	NH-5	caprine	III	negative	negative	n.p.	
Rudabánya	K-1	caprine	II	negative	negative	n.p.	
Rudabánya	K-2	caprine	II	negative	negative	n.p.	
Rudabánya	K-3	caprine	II	negative	negative	n.p.	
Rudabánya	K-4	caprine	II	negative	negative	n.p.	
Rudabánya	K-5	caprine	II	negative	negative	n.p.	
Rudabánya	K-6	caprine	II	negative	negative	n.p.	
Rudabánya	K-7	caprine	II	negative	negative	n.p.	
Rudabánya	K-8	caprine	II	negative	negative	n.p.	

Rudabánya	K-9	caprine	II	negative	negative	n.p.	
Rudabánya	K-10	caprine	II	negative	negative	n.p.	
Rudabánya	K-11	caprine	II	negative	negative	n.p.	
Rudabánya	K-12	caprine	II	negative	negative	n.p.	

Table S1: Detailed background information of individual samples used for the epidemiological investigation of misa-, sisa-, and tusaviruses including the results of MiSiTuV-NSP screening and MiSiTuV-VP typing nested PCR reactions. PCR1: 1st round of the nested PCR, PCR2: 2nd round of the nested PCR; ID: identification marks. Age group I: < 2-month-old animals, Age group II. 2-12-month-old animals, Age group III: >12-month-old animals. n.p.: not performed (only when the prior MiSiTuV-NSP screening was negative). Accession numbers of the determined complete coding sequences of misa-, sisa- and gisavirus are marked with an #. NSP: non-structural protein encoding genome region, VP: viral capsid protein encoding genome region. CDS: complete coding sequence
